# Soft computing techniques for predicting the properties of raw rice husk concrete bricks using regression-based machine learning approaches

**DOI:** 10.1038/s41598-023-41848-1

**Published:** 2023-09-04

**Authors:** Nakkeeran Ganasen, L. Krishnaraj, Kennedy C. Onyelowe, George Uwadiegwu Alaneme, Obeten Nicholas Otu

**Affiliations:** 1 Department of Civil Engineering, SRM Institute of Science and Technology, Kattankulathur, Chengalpattu, Tamil Nadu 603203 India; 2 Department of Civil Engineering, Michael Okpara University of Agriculture, Umudike, Nigeria; 3 Department of Civil Engineering, Kampala International University, Kampala, Uganda; 4 Department of Civil Engineering, University of Cross River State, Calabar, Nigeria

**Keywords:** Engineering, Materials science

## Abstract

In this study, the replacement of raw rice husk, fly ash, and hydrated lime for fine aggregate and cement was evaluated in making raw rice husk-concrete brick. This study optimizes compressive strength, water absorption, and dry density of concrete brick containing recycled aggregates via Response Surface Methodology. The optimized model's accuracy is validated through Artificial Neural Network and Multiple Linear Regression. The Artificial Neural Network model captured the 100 data's variability from RSM optimization as indicated by the high R threshold- (R > 0.9997), (R > 0.99993), (R > 0.99997). Multiple Linear Regression model captured the data's variability the decent R^2^ threshold confirming- (R^2^ > 0.9855), (R^2^ > 0.9768), (R^2^ > 0.9155). The raw rice husk-concrete brick 28-day compressive strength, water absorption, and density prediction were more accurate when using Response Surface Methodology and Artificial Neural Network compared to Multiple Linear Regression. Lower MAE and RMSE, coupled with higher R^2^ values, unequivocally indicate the model's superior performance. Additionally, employing sensitivity analysis, the influence of the six input parameters on outcomes was assessed. Machine learning aids efficient prediction of concrete's mechanical properties, conserving time, labor, and resources in civil engineering.

## Introduction

Globally, developing urban development and improved living standards due to technological advancements have necessitated a significant construction industry's use of natural resources, resulting in insufficiency^1^. Alternatively, the environmental impact of disposing of industrial and agricultural waste has been a persistent issue.^2^. Fly ash is a major solid waste from the coal industry^3^. While each year, millions of tons of rice husk are produced as agricultural waste material. According to the United States Department of Agriculture, 513.02 million metric tons of rice were produced globally in 2021^4^. Due to the enormous rice production, a massive amount of rice husk is also produced worldwide. Fly ash generation data for 2020–2021 has been received from 202 (Two Hundred and Two) coal/lignite thermal power stations in India^5^.

The construction industry is highly dependent on ordinary Portland cement (OPC). Raw materials for cement production usually consist from non-renewable sources due to their high expense of the environment. For the predictable future, the availability of these materials is in problem^6^. Conventional OPC cement manufacturing is also a major contributor to greenhouse gas emissions and global warming. Cement production consumes approximately 4 giga joule (GJ) of energy and 1.7 tons of raw materials, and releases approximately 0.73–0.99 tons of CO_2_ per ton^7^. FA (Fly Ash)^8^ and hydrated lime^9^ have been used as supplementary cement materials for the last decades.

Green building rating (GBR) programs currently evaluation of buildings environmental performance in a variety of subcategories, including the material used to build them. Sustainability in the construction industry is more concerned with issues such as carbon dioxide emissions, aggregates, fillers, and demolition waste used in the production of concrete^10^. Rice husk (RH) as cement replacing material^11^, insulation material^12^, marking the concrete brick and tiles^13^, board production^14^, ceramic tiles^15^ and replacement of aggregate^16^, these concretes are used to build affordable housing^17^.

Cement and aggregates have been substituted with industrial and agro-industrial wastes to produce cheaper concrete brick while preserving natural resources and properly managing solid waste. The hemp used in concrete brick^18^. Similar concrete brick was made from rice husk (RH), fly ash (FA), and hydrated lime (HL) were among the industrial and agricultural wastes that had potential substitutes for aggregate and cement.

Cement, water, and partial replacement of fine aggregate incorporate in the making concrete brick. After 28 days of curing, concrete has reached its maximum final strength, which is a standard for calculating its strength over time. Concrete's compressive strength, water absorption, and density are all critical properties that are used in the wall structure design in construction^19^. Recently, statistical methods, analytic modelling and the use of artificial intelligence (AI) have been used to predict and optimize material, mix combination and the strength of concrete that incorporates a wide range of different ingredients^20–22^. When reviewing the literature, researchers need to know the best parameter for achieving a wide range of mechanical and physical characteristics. As a result, other techniques should be employed to improve the ratios in the mixtures^23^. Different techniques for enhancing performance are employed to determine these variables impact precisely and to obtain the best-expected concrete shown in Table 1. In this study, RSM, ANN, and MLR are just three of the approaches used for prediction. RSM, ANN, and MLR-based mathematical models are accessible and simple, even for practitioners with limited knowledge of with regard to modelling and computation.

**Table 1:** Models used for prediction.

Models^(a)^	Material	Properties^(b)^	Days	Results	References
MNR and ANN	Recycled aggregate concrete (RAC)	CS, EM, FS and TS	28th	Comparison of models	^24^
ANN, fuzzy TSK, SVR and RBFNN	Recycled aggregate concrete (RAC)	EM	28th	Prediction	^25^
ANN	Agricultural wastes concrete	TS	28th	Prediction	^26^
RSM and ANN	Recycled coarse aggregate concrete	CS	56th	Comparison of models	^27^
ANN and SVM	Conventional concrete	CS	28th	Prediction	^23^
ANN	Conventional concrete	CS	28th	Prediction	^28^
ANN	Construction and demolition waste (CDW) concrete	CS	91th	Prediction	^29^
ANN and ANFIS	Normal concrete (NC) and high-performance concrete (HPC)	CS	–	Prediction	^30^
MRA and ANN	High performance concrete	CS	90th	Comparison of models	^31^
ANN	High-performance concrete (HPC)	CS and TS	28th	Prediction	^32^
ANN	Replacement of cement (metakaolin)	CS	28th	Prediction	^33^
ANN	Recycled aggregate concrete (RAC)	CS	–	Prediction	^34^
RSM	Hybrid concrete (HC)	CS, WA	28th	Prediction	^35^
RSM	Ultra-high performance concrete	Shrinkage	–	Optimization	^36^
RSM	Ultra-high performance concrete	CS	28th	Prediction	^37^
RSM	Self-consolidating concrete	CS, fresh properties	–	Optimization	^38^
ANN and RSM	Conventional concrete	CS	28th	Comparison of models	^39^
ANN and MLR	Recycled aggregate concrete (RAC)	CS	28th	Comparison of models	^40^
ANN and MLR	Recycled aggregate concrete (RAC)	CS, TC, FS	28th	Comparison of models	^41^

^(a)^*MNR* multiple nonlinear regression, *ANN* artificial neural networks, *MRA* multiple regression analysis, *SVM* support vector machine, *ANFIS* adaptive neuro-fuzzy inference system, *RBFNN* radial basis function neural network, *SVR* support vector regression, *RSM* response surface methodology.

^(b)^*CS* compressive strength, *TS* tensile strength, *FS* flexural strength, *EM* elastic modulus, *WA* water absorption.

Fast and accurate performance is obtained by using the RSM statistical method with a small number of experiments in a short period of time^42^. Since 1999, the RSM method was used to predict future outcomes and the properties of high-performance concrete (HPC). Despite the widespread use of this strategy in the design and optimization of trials, the concrete industry has had limited use of this strategy^43^. The RSM approach examined the influence of various characteristics of self-compacting concrete's fluidity, rheology, and strength. The RSM method was used to examine the effects of properties such as superplasticizer, drying time, shrinkage, compressive strength, and cost were taken into consideration when determining the optimal parameters for these samples^44^. The RSM method was used to study the impact of FA, foam volume, and the pozzolan-cement ratio on the compressive strength of Foarm concrete FC^45^. As measured by the correlation coefficient of their RSM model, the RSM improves concrete with FA and metakaolin that has high CS while reducing water sorptivity, absorption, and chloride penetration^46^. Research that makes concrete can use the model generated by this method.

ANN have been put to use in a wide range of civil engineering applications, including structural damage detection, identification of the structural system, models for predicting foundation settlement and proportioning and strength prediction of concrete mixtures, as well as monitoring of material behavior and groundwater^47, 48^. According to a recent review, artificial neural network (ANN) models are increasingly being used to predict concrete properties^49, 50^. These studies include creep strain compressive strength, tensile strength, flexural strength, chloride concentration resistivity, and fresh properties^51^. In addition, ANN predicted outcomes have been used in several studies the strengths of various types of concrete^52^. Recycling aggregate concrete has a high compressive strength, which was predicted with precision by the model^53^. Therefore, ANNs were used to predict strength. According to the findings of the study, it was concluded that the ANN model with the Levenberg–Marquardt training function was the most accurate predictive tool^54^. Systems that use fly ash instead of sand have been modelled using neural network models^55^. In recent years, studies on improving the content of material have made use of these approaches. Recent research indicates that ANN, RSM models are gradually being upgraded out conventional MLR for predicting specific characteristics.

This effort included partial sand replacement with RH and replacement cement with fly ash and hydrated lime and measured compressive strength, water absorption and density of R. Similar studies are fly ash mortar and measured compressive strength and water absorption^56^, and another study, fine aggregate, replaced and measured compressive strength, water absorption and density of RHCB to simulate system^57^. In this research is being carried out to model the properties of rice husk concrete brick (RHCB) as input of Raw rice husk, Fly ash, Hydrated lime and output as compressive strength, water absorption and density using Response Surface Methodology, artificial neural networks (ANNs), and multi-linear regression (MLR). In this work, the performance of RHCB was predicted using RSM, ANN and MLR and compared to experimental data results of compressive strength, water absorption and density.

### Conceptual framework

**Figure 1:**
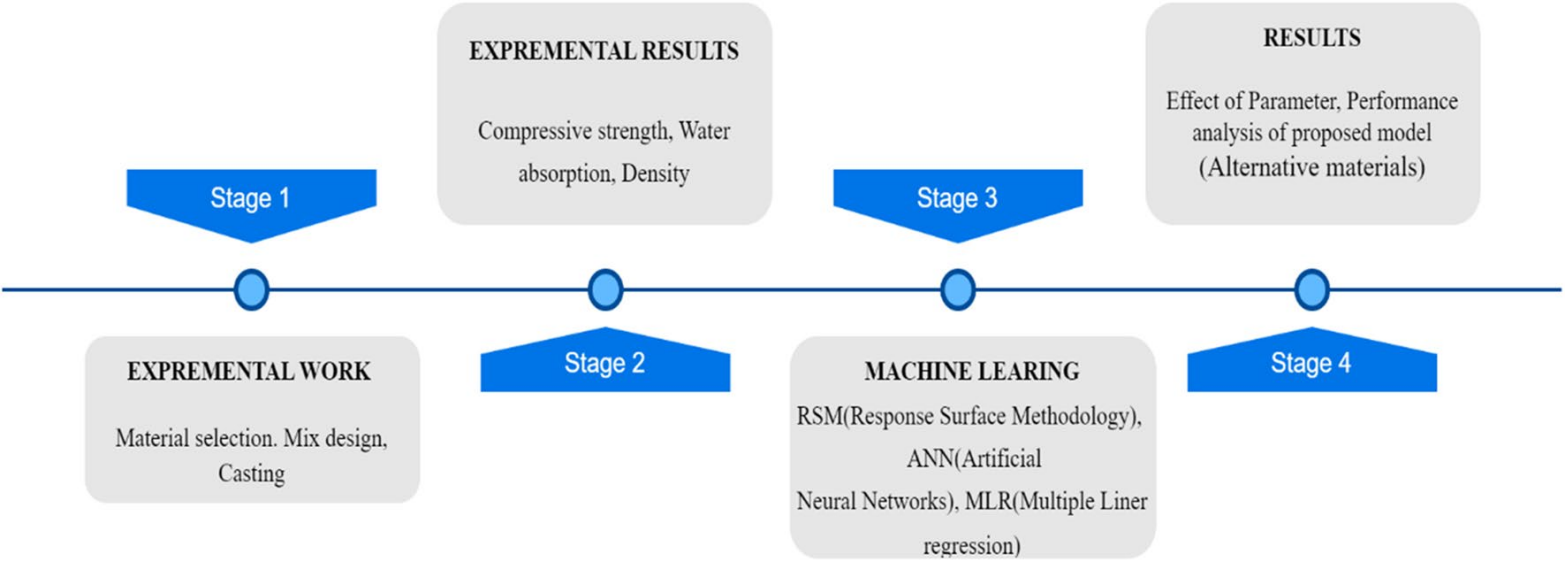
Main stages of the procedure.

A descriptive statement, experimental data, objective functions (ML, RSM, ANN, MLR), and statistical R^2^ are the four major stages of the proposed procedure for mathematically analyzing RHCB. Based on the requirement in the manuscript, the first step is to create a descriptive statement. Next, the design variables would have determined, and data gathered through experiments and observations. The third stage entails creating machine-learning objectives (RSM, ANN, MLR). Finally, optimization algorithms are applied to the obtained objective functions in the fourth and final stage. In Fig. 1, a simplified version of this procedure is shown.

## Materials and methods

**Figure 2:**
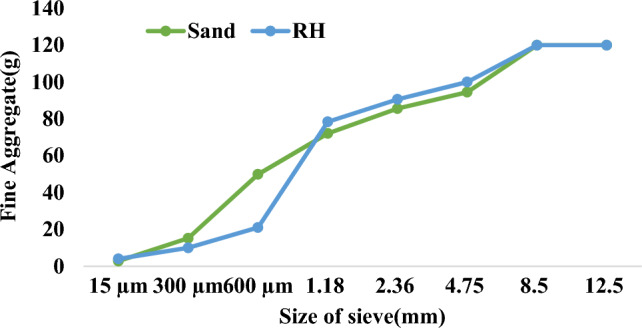
Sieve analysis of fine aggregate and RH.

Agricultural waste is used in concrete brick-like sawdust, coconut-shell, and peanut shell using a mix design of 1:6 (cement: sand)^58^. Similarly, Table 2 shows mix design of RHCB in the article. Hydrated lime, fly ash, and cement were all used as binder in the mix of RHCB. Figure 2 summarizes the sieve analysis of fine aggregate and raw RH used in this experiment. Compressive strength, water absorption, and density are listed in Table 3 as a result of the testing. For machine learning algorithms necessitate multiple input variables to generate the targeted output variable. Within the realm of concrete construction and analysis, paramount significance is attributed to parameters such as compressive strength, water absorption, and dry density. Leveraging the input factors and data instances, a robust and efficacious model is formulated to encompass the incorporation of raw agricultural waste within concrete compositions.

**Table 2:** Mix ratio and material combination.

Mix design	Mix ratio	Cement (g)	Sand (g)	RH (g)	FA (g)	HL (g)	W/C
RH1	1:5:0.1	1000	5419	98	0	0	0.4
RH2	1:4:0.2	1000	4335	196	0	0	0.4
RH3	1:3:0.3	1000	3251	295	0	0	0.4
RH4	1:2:0.4	1000	2147	392	0	0	0.4
RH5	1:1:0.5	1000	1087	490	0	0	0.4
RH6	1:0:0.6	1000	0	588	0	0	0.4
RH7	1:6:0	1000	6000	0	0	0	0.4
RHFH1	1:5:0.1	700	5419	98	200	100	0.4
RHFH2	1:4:0.2	700	4335	196	200	100	0.4
RHFH3	1:3:0.3	700	3251	295	200	100	0.4
RHFH4	1:2:0.4	700	2147	392	200	100	0.4
RHFH5	1:1:0.5	700	1087	490	200	100	0.4
RHFH6	1:0:0.6	700	0	588	200	100	0.4
RHFH7	1:8.57:0	700	6000	0	200	100	0.4

**Table 3:** Compressive, water abortion, and density.

Mix design	Compressive strength (MPa)	Water absorption (%)	Density (kg/m^3^)
RH1	5.9	190	3.59
RH2	4.6	205	2.87
RH3	3.89	250	2.43
RH4	3.4	270	2.16
RH5	2.9	300	2.02
RH6	2.2	340	1.75
RH7	6.65	0	4.72
RHFH1	5.82	187	3.47
RHFH2	4.5	213	2.62
RHFH3	3.8	254	2.26
RHFH4	3.37	277	2.04
RHFH5	2.89	309	1.96
RHFH6	2.21	350	1.64
RHFH7	6.25	0	4.4

### Methods

The utilization of response surface methodology (RSM) enables the optimization of concrete brick properties, such as compressive strength, water absorption, and dry density, by systematically exploring the relationship between input variables. Meanwhile, the incorporation of artificial neural network (ANN) validation ensures the reliability and generalization of the developed model, validating its predictive capabilities against unseen data.

#### Response surface methodology (RSM)

RSM is a collection of procedures for analyzing data for examining and modelling that there are functional connections between the input variables (x) and the desired response (y)^59^. The RSM is a method for analyzing the results of experiments. Calculating the R^2^, R^2^ adjusted, and R^2^ predicted quantity determined the model’s significance level. The calculated F-quantity is used to determine the influence of the factors on the measured results. The greater the F-quantity for a parameter, the more significant the experiment's results will be as a result of that parameter. The P-quantity tells readers whether or not the model's results are effective. A P-quantity of less than 0.05 can identify an important model or set of parameters.

This is an RSM polynomial model, where x and y are the input and output variables, respectively. Equations (1) and (2) express the polynomial model, respectively. Design expert v10.0.1 software was used to perform RSM modelling on historical data. The advantage of using historical data design (HDD) is that it removes the need to stick to a specific experimental design and allows for the study to accept data of any size to be input^60, 61^.

1 $${\varvec{y}}=\boldsymbol{ }{{\varvec{\beta}}}_{{\varvec{o}}}+{\sum }_{{\varvec{i}}=1}^{{\varvec{k}}}{{\varvec{\beta}}}_{{\varvec{i}}}{{\varvec{x}}}_{{\varvec{i}}}+{\varvec{\varepsilon}}$$

2 $${\varvec{y}}=\boldsymbol{ }{{\varvec{b}}}_{{\varvec{o}}}+\sum_{{\varvec{i}}=1}^{{\varvec{n}}}{{\varvec{b}}}_{{\varvec{i}}}{{\varvec{x}}}_{{\varvec{i}}}+{\sum }_{{\varvec{i}}=1}^{{\varvec{n}}}{{\varvec{b}}}_{{\varvec{i}}{\varvec{i}}}{{\varvec{x}}}_{{\varvec{i}}}^{2}+{\sum }_{{\varvec{i}}=1}^{{\varvec{n}}}{{\varvec{b}}}_{{\varvec{i}}{\varvec{j}}}{{\varvec{x}}}_{{\varvec{i}}}{{\varvec{x}}}_{{\varvec{j}}}+{\varvec{\varepsilon}}$$

where Y represents the predicted response function, ${{\varvec{\beta}}}_{{\varvec{i}}}$ represents the intercept, ${{\varvec{b}}}_{{\varvec{i}}}{,\boldsymbol{ }{\varvec{b}}}_{{\varvec{i}}{\varvec{i}}}$ represent linear effect coefficients, ${{{\varvec{x}}}_{{\varvec{i}}},{{\varvec{x}}}_{{\varvec{j}}},{\varvec{x}}}_{{\varvec{i}}}^{2}$ represent quadratic effect coefficients, and ${{\varvec{\beta}}}_{0},\boldsymbol{ }{{\varvec{b}}}_{0}$ represents the interaction effect coefficient, n is number of data, K is total number of data.

#### Artificial neural networks (ANN)

**Figure 3:**
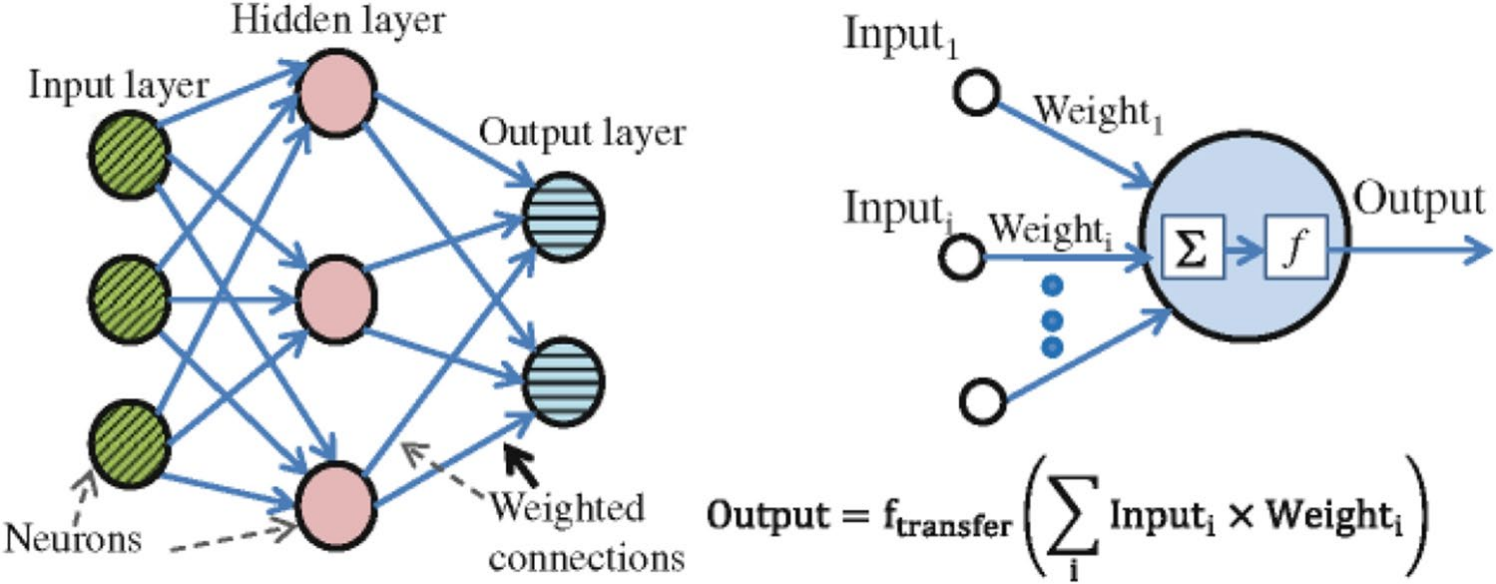
Regular neural model of ANN.

The human brain's functional neural features can be used to generate mathematical and numerical models. With processing and representation of data, the ANN is an input and output is linked by various data structures that are linked together in a statistical model with which has multiple neurons capable of large computations^62, 63^. An artificial neural network model to predict the desired output from a given input can be trained. The output of each layer will go to ensure that the layer's input is compatible with the transfer function with the desired outcome regular ANN showed in Fig. 3. The function of transmission can be either linear or nonlinear in its behavior. Most of the time, the hidden layers are nonlinear, while the output layers are predicted output is then processed by the output layer^64, 65^. Equation (3) can be used to summarize all of ANN’s processes. A single output is created by adding bias (b) to the sum of the individual outputs. ANN model was implemented this research using MATLAB R2020b software.

3 $$\theta ={f}_{sig}\{{b}_{o}+\sum_{j=1}^{n}\left[{w}_{j}\times {f}_{sig}({b}_{hj}+{\sum }_{i=1}^{m}{w}_{ij}{\delta }_{i}\right]$$

#### Multiple linear regression (MLR)

Multiple engineering disciplines have relied on this model’s ability to establish linear relationships between variables. Modeling the relationship between the dependent variable and more than one independent variable is accomplished using MLR^66, 67^. Minimizing the difference between variables- the dependent and independent is an important principle behind MLR. The regular mathematical form of the MLR model is as follows.

4 $$Y={c}_{0}+{c}_{1}{X}_{1}+{c}_{2}{X}_{2} +...+{c}_{n}{X}_{n}+\beta$$

As shown in Eq. (4), the dependent variable is *Y*, *c*_0_ is the intercept, *c*_1_ to *c*_n_ are the coefficients associated with independent variables, *X*_1_ to *X*_n_ are independent variables, and is the error related to the predictor.

## Results

### Compressive strength

Predictable RSM methods were used to predict the 28-day compressive strength design type in 14 different experimental combination results. The quadratic model of design is employed in the process. The rationale for opting for a quadratic model over other models in Response Surface Methodology (RSM) is rooted in its capacity to account for nonlinearity, curvature, and complex interactions among variables, thereby ensuring a more accurate representation of the underlying system. This choice is pivotal for precise optimization and predictive capabilities. A summary of RSM's design can be found in Table 4.

**Table 4:** Design of RSM.

File version	Type of study	Style of design	Model	Subtype	Runs	Blocks
13.0.11.0	Response surface	Spreadsheet (used-defined)	Quadratic	Randomized	14	No blocks

Table 5 showed that ANOVA provides the sum of squares, the df, the mean square, the F-quantity, and the p-quantity at the 5% level of significance. All three responses had R^2^ ≥ 0.9923. To avoid an unnecessarily large increase in the adjusted R^2^, this term refers to an adjustment to the R^2^. R^2^ that is within 0.1 of the adjusted version of the R^2^ would be preferable^68^. This was the situation in question in this research. In addition, if the p-quantity is greater than 0.05, the model is statistically significant^69, 70^. The research implications of the ANOVA outcomes lie in the *p* value and F-value results. The *p* value indicates statistical significance, helping accept or reject hypotheses. The F-value signifies the variance explained by the model, guiding the understanding of relationships among variables, thus influencing subsequent analyses and conclusions.

**Table 5:** Analysis of variance (RSM-ANOVA) using a quadratic model.

Source	Sum of squares	df	Mean square	F-value	*p* Value	
Model	28.69	8	3.59	81.1	< 0.0001	Significant
A-cement	0.0507	1	0.0507	1.15	0.3331	
B-sand	0.0111	1	0.0111	0.25	0.6378	
C-rice husk	0.0105	1	0.0105	0.24	0.6468	
AB	0.0195	1	0.0195	0.44	0.5364	
AC	0.0232	1	0.0232	0.52	0.5016	
BC	0.0152	1	0.0152	0.34	0.5826	
B^2^	0.0149	1	0.0149	0.34	0.5863	
C^2^	0.0156	1	0.0156	0.35	0.5788	
Residual	0.2212	5	0.0442			
Cor total	28.91	13				
SD	0.2103		R^2^	0.9923	Adeq precision	25.8
Mean	4.17		Adjusted R^2^	0.98		
C.V.%	5.04		Predicted R^2^	0.92		

**Figure 4:**
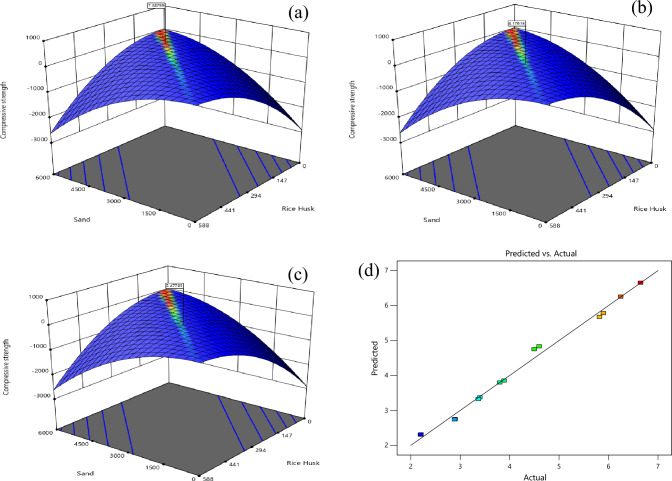
Maximum Compressive strength (**a**), optimum compressive strength (**b**) and minimum Compressive strength (**c**) and actual and predicted (**d**) value from RSM. Note* compressive strength (MPa), Sand (g), Rice husk (g).

Three-dimensional RSM plots are shown in Fig. 4 displays the scatter plots for all of the data collected. Parity and three-dimensional plots of dependent variables are depicted in Fig. 4. The Fig. 4 parity and 3D plots of compressive strength reveal that the factors A and B interact strongly. The sand, raw rice husk, and cement formed a strong bond. When plotting the predictions, it was discovered that they are very in close proximity to the diagonal. As a result, the RSM model accurately predicted the outcome, with a balanced distribution of data points on either side of the diagonal. This finding demonstrates a lack of bias in over-or under-prediction. As a major drawback of RSM, estimating one factor influences its analysis of another factor, which is a major issue. According to the actual and predicted response quantity, it is clear that the responses are very close together. The residual plots showed no significant deviation from normality, indicating that the chosen model effectively predicted the material’s strength and interaction^71^.

The equations for the RSM method model for CS can be found in Eq. (5) and respectively.

5 $$\begin{aligned} CS & = 2924.50029 + 0.012017C + 0.936817D + 9.80967E \\ & \quad - 1.78E - 06C \times D - 0.00002C \times E0.00157D \\ & \quad \times E - 0.000075D^{2} - 0.008218E^{2} \\ \end{aligned}$$

**Figure 5:**
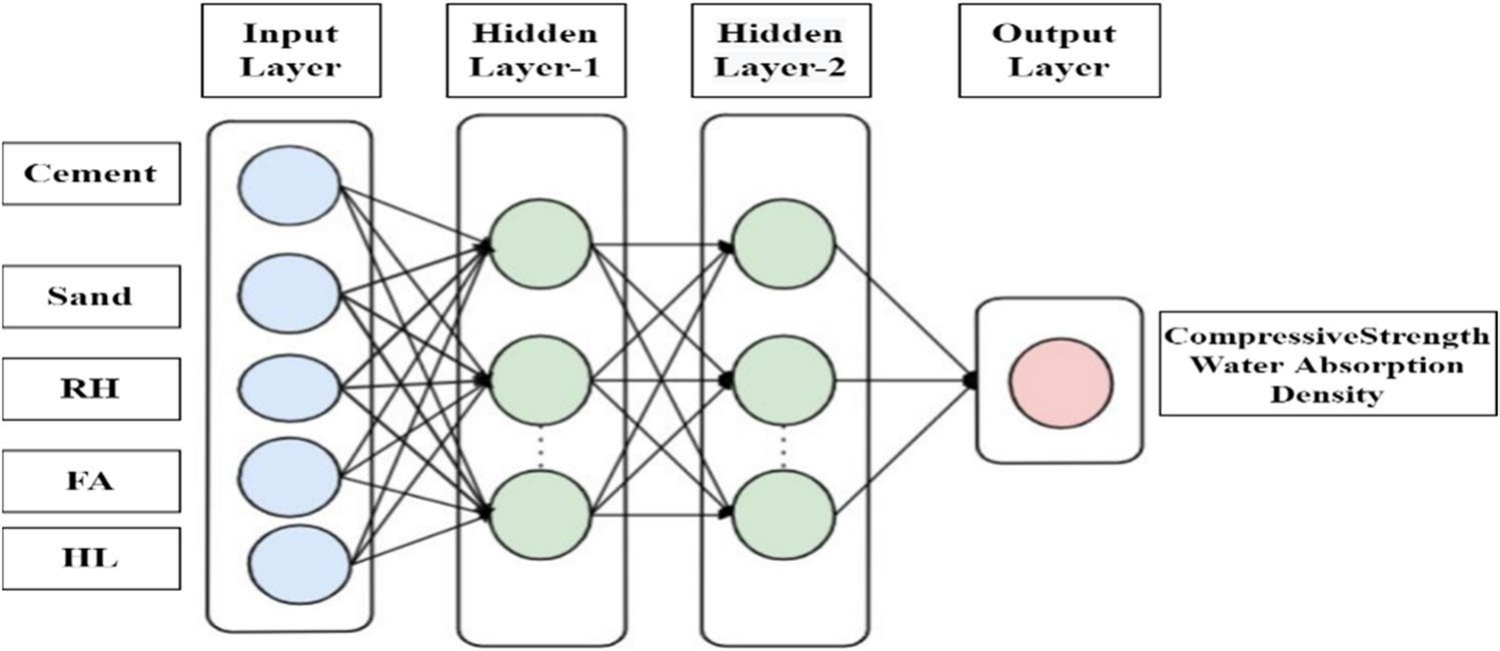
Architecture of ANN utilized for prediction.

**Figure 6:**
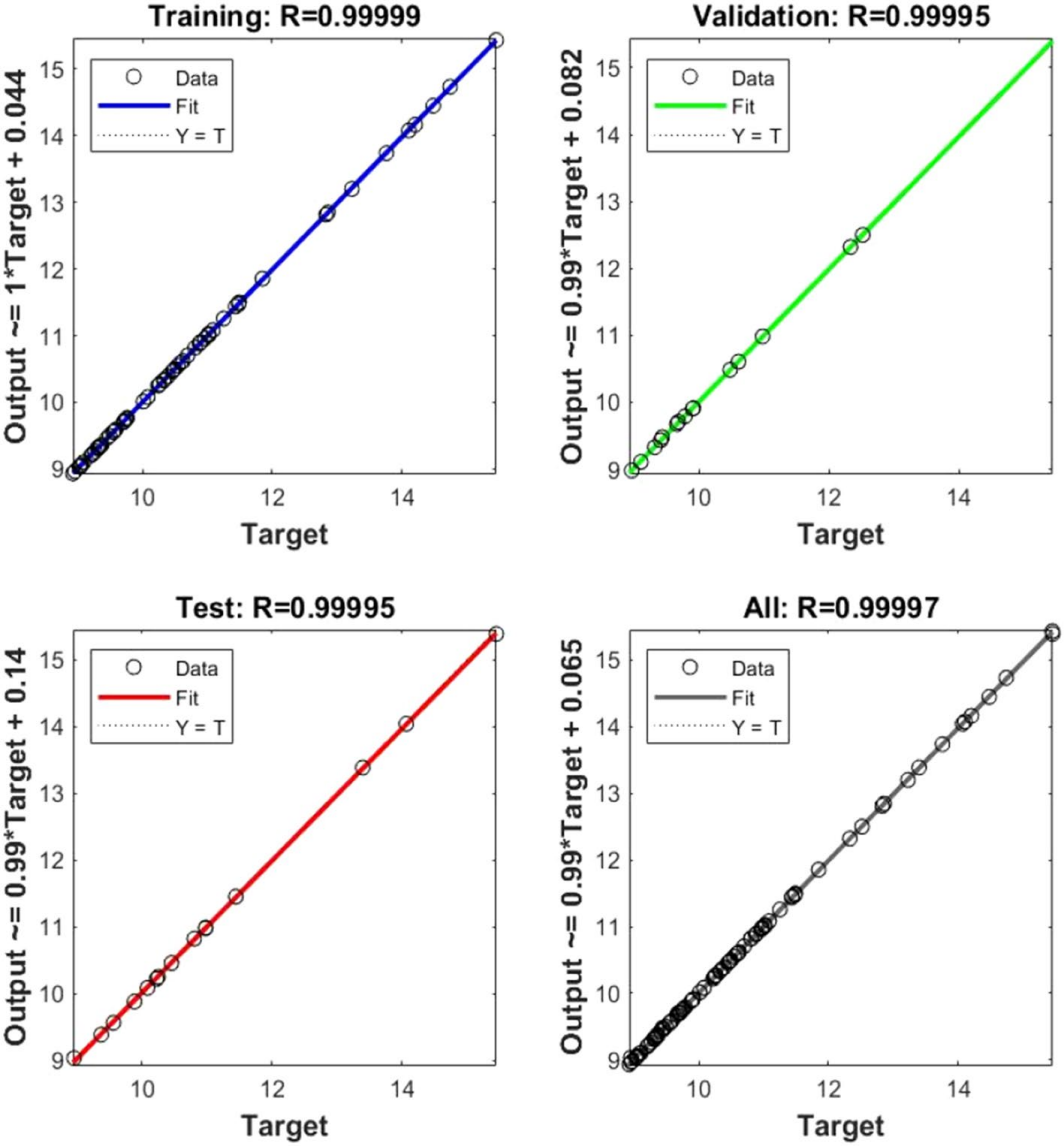
Compressive strength prediction in ANN.

In Eq. (5), where the C stands for fly ash, the D is the hydrated lime, and the E is the raw rice husk, until otherwise stated. The RSM was done before to materials change of RHA with a cement replacement show a CS of RHCB. In this research, the maximum CS was calculated to be 7.34759 MPa, the minimum was calculated to be 2.47793, and the target was calculated to be 4.17615 according to this diagram (Fig. 3). This is referred that the estimated value increases. The closeness of the actual and predicted responses can be seen in the response values, which compare the actual with the predicted. The residual plots showed no significant deviation from normality; the plots clearly show in predicting the strength and interaction of the materials, that model was accurate. The architecture of ANN based on the data provided is shown in Fig. 5 used three different models. The proposed ANN regression plots are also shown in Fig. 6. This study employed MATLAB to train an artificial neural network (ANN) with 5 input layers, 1 hidden layer featuring 10 neurons, and 1 output layer. For MATLAB analysis, 75% (75 data) were allocated for training, 15% (15 data) for testing, and 15% (15 data) for validation. The R quantities that were used to train, validate, and test the model are all above 0.99997. And they can be approximated to unity, which stands for one hundred percent (Fig. 6). An R-value greater than 0.9 indicates that the model can be relied upon to provide an accurate prediction.

**Figure 7:**
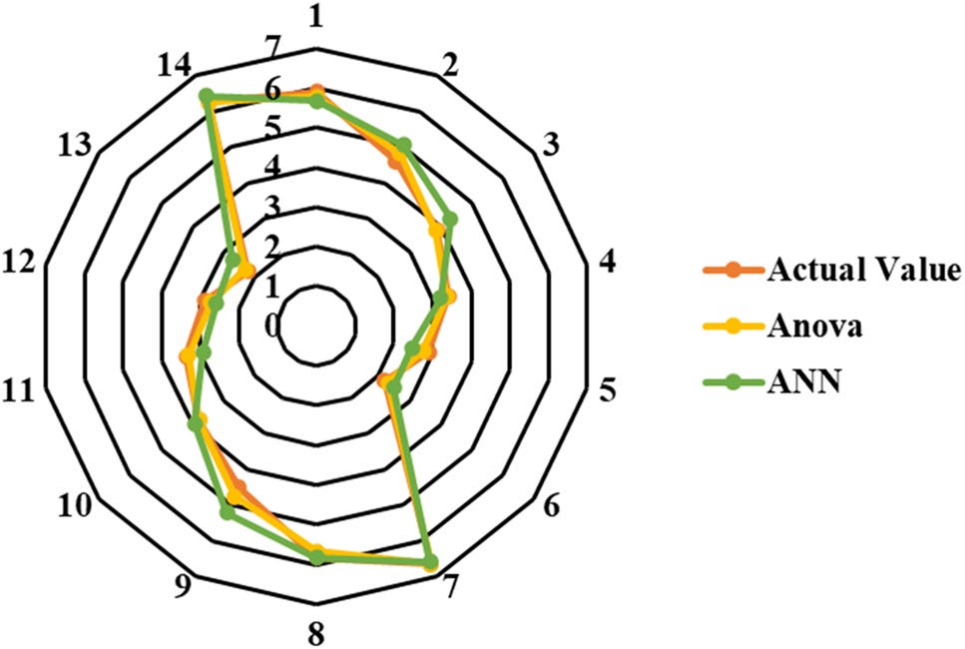
Compressive strength prediction and actual value.

**Figure 8:**
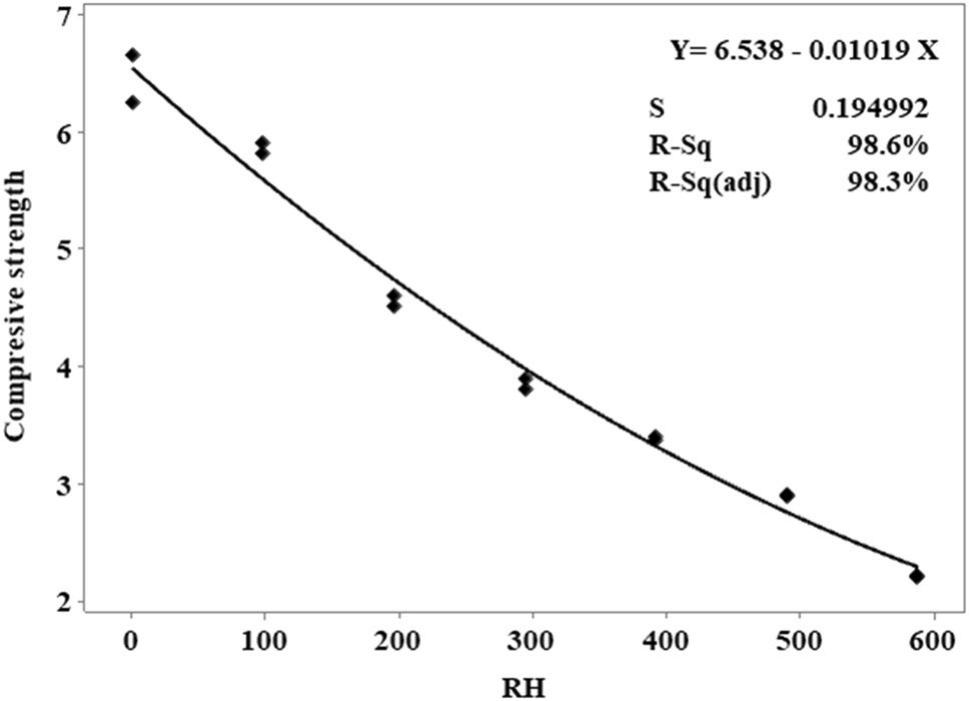
Compressive strength prediction in MLR. Note* compressive strength (MPa), RH (g).

Again, the results in both models and the predictions match the results observed in the experiments, as seen in the Figs. 7 and 8 below. With graphic depiction of a radar system Fig. 7 shows the model’s results compared to the test’s results. To put it another way, the values that were estimated by the test results are very close to the actual values. ANOVA was also used to test the significance of compressive strength models and model terms at a 95% confidence level. After analyzing the ANN method and the ANOVA method for both of the strength values, it was discovered that the RSM and ANN method was more appropriate for estimating the results. Table 6 results are shown of the experiments and the models used to estimate the CS.

**Table 6:** Predicted value of ANN and ANOVA.

Mix design	Actual value	Predicted value	
		ANOVA	ANN
RH1	5.9	5.78	5.66676
RH2	4.6	4.83	5.05961
RH3	3.89	3.85	4.32499
RH4	3.4	3.37	3.21051
RH5	2.9	2.75	2.46273
RH6	2.2	2.3	2.48094
RH7	6.65	6.65	6.58293
RHFH1	5.82	5.67	5.83338
RHFH2	4.5	4.75	5.20212
RHFH3	3.8	3.8	3.93923
RHFH4	3.37	3.32	2.92773
RHFH5	2.89	2.74	2.59316
RHFH6	2.21	2.31	2.69983
RHFH7	6.25	6.25	6.43073

Predicted outcomes differ greatly from one another compressive strength values from a comparison between the MLR model and the results obtained in the laboratory. Figure 8 indicates that the lower RH led to the higher compressive strength. Decreasing in RH replacement increases the compressive strength among the five independent variables. In Fig. 8, the data points are fitted line plots. The results of the analysis MLR 0.9855 R^2^ predict the accurately and quickly of 98.55%.

### Water absorption

There were 14 separate experiments used to develop the 28-day water absorption. A quadratic model is used to create it. RSM-ANOVA results of RHCB water absorption can be seen in Table 7 for more information.

**Table 7:** RSM-AVOVA of water absorption.

Source	Sum of squares	df	Mean square	F-value	*p* Value	
Model	152,200	8	19,018.93	2660.97	< 0.0001	Significant
A-C	0.4246	1	0.4246	0.0594	0.8171	
B-D	158.45	1	158.45	22.17	0.0053	
C-E	157.42	1	157.42	22.02	0.0054	
AB	0.4631	1	0.4631	0.0648	0.8092	
AC	0.0402	1	0.0402	0.0056	0.9432	
BC	158.65	1	158.65	22.2	0.0053	
B^2^	159.48	1	159.48	22.31	0.0052	
C^2^	157.73	1	157.73	22.07	0.0053	
Residual	35.74	5	7.15			
Cor total	152,200	13				
SD	2.67	R^2^	0.9998	Adeq precision	163.8869	
Mean	224.64	Adjusted R^2^	0.9994			
C.V.%	1.19	Predicted R^2^	0.9972			

**Figure 9:**
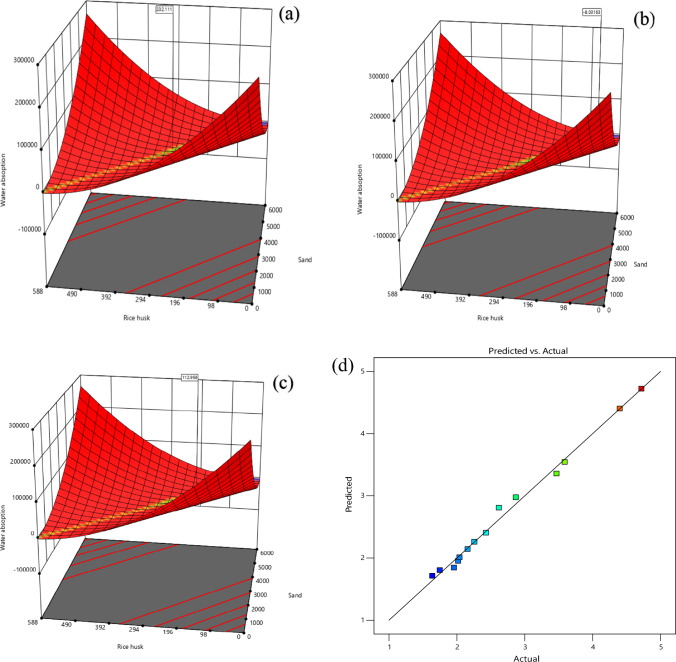
Maximum water absorption, optimum water absorption and minimum water absorption and actual and predicted value from RSM. Note*: Water absorption (%), Rice husk (g), Sand (g).

According to the model's F-quantity of 2660.97, it is significant. If noise causes an F-quatity to be large, the probability is less than 0.01%. A model term with a P-quantity of less than 0.0500 indicates significance. Among the relevant model terms here are B, C, BC, B^2^, and C^2^. The model terms are insignificant if their values are greater than 0.1000. Reducing the number of unimportant model terms (excluding those necessary to support hierarchical structure) can improve the models. Because the predicted R^2^ of 0.9972 and the adjusted R^2^ of 0.9994 are within 0.2 standard deviations of one another, someone can conclude that the two are fairly well matched. Adeq Precision calculates SNR (signal to noise ratio). More than a four-to-one ratio is preferred. The signal-to-noise ratio of 163.887 indicates that next setup is working properly. This model can be used to find an efficient way around a design's various options and possibilities (Table 7). The maximum water absorption was calculated to be 232.111, minimum was calculated to be 112.956, and target was calculated to be 8.03163 according to the Fig. 9.

The closeness of the actual vs. predicted responses can be seen in the response values, which compare the actual with the predicted. The residual plots showed no significant deviation from normality; the plots clearly show in Fig. 9d that the chosen model accurately predicted the absorption of water and the interaction of the materials used in the experiment. Equation (6) is the model equations derived using the RSM method for water absorption.

6 $$\begin{aligned} Water\;absorption & = 299379 - 0.052192{\text{C}} - 96.23521{\text{D}} - 994.85274{\text{E}} \\ & \quad + 8.69{\text{E}} - 06{\text{C}} \times {\text{D}} + 0.000027{\text{C }} \times {\text{E}} + 0.160183{\text{D}} \\ & \quad \times {\text{E}} + 0.007723{\text{D}}^{2} + 0.827119{\text{E}}^{2} \\ \end{aligned}$$

**Figure 10:**
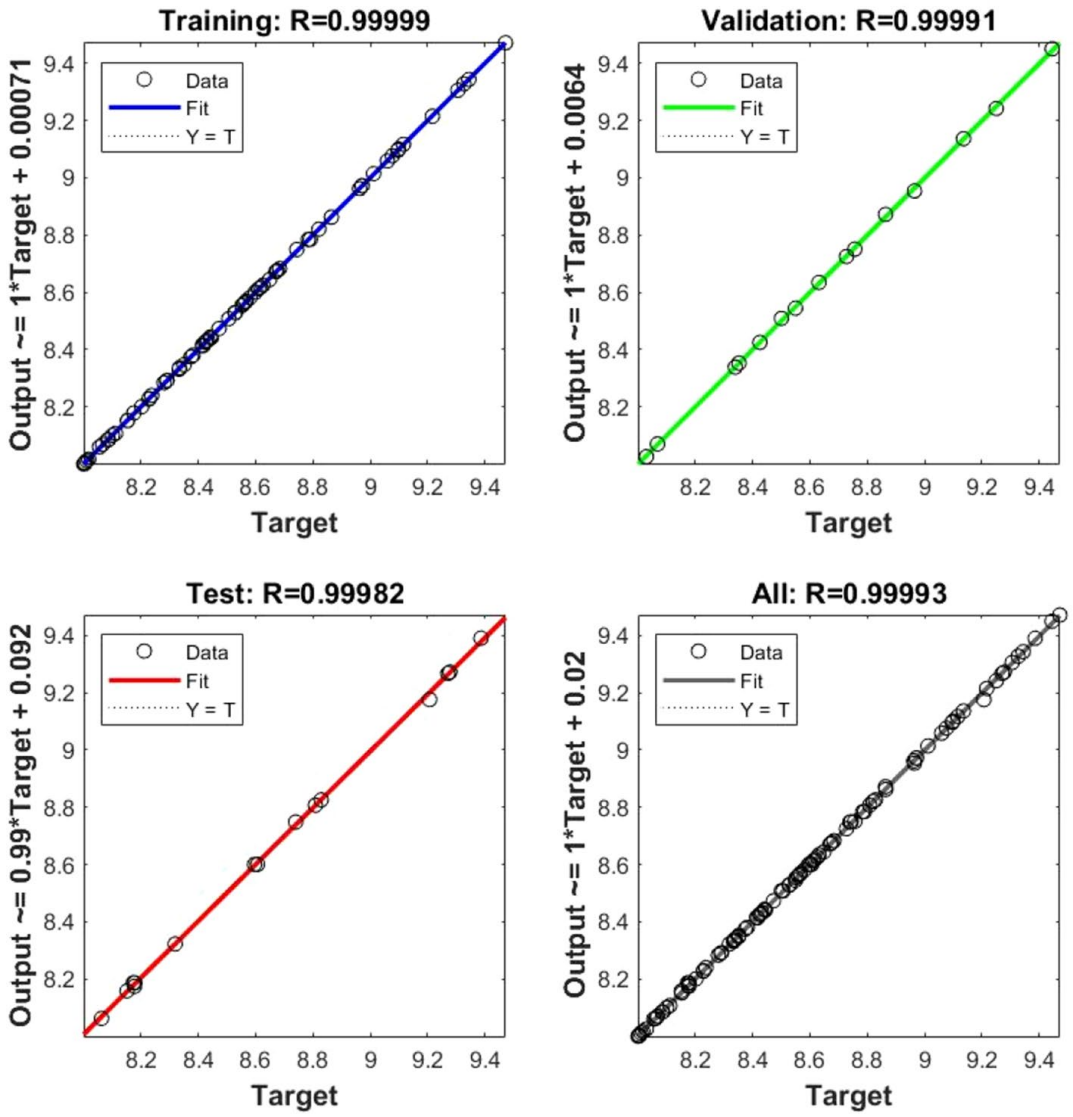
Water absorption with ANN.

Figure 10 shows the R correlation coefficient for water absorption, resulting from an ANN analysis.

**Figure 11:**
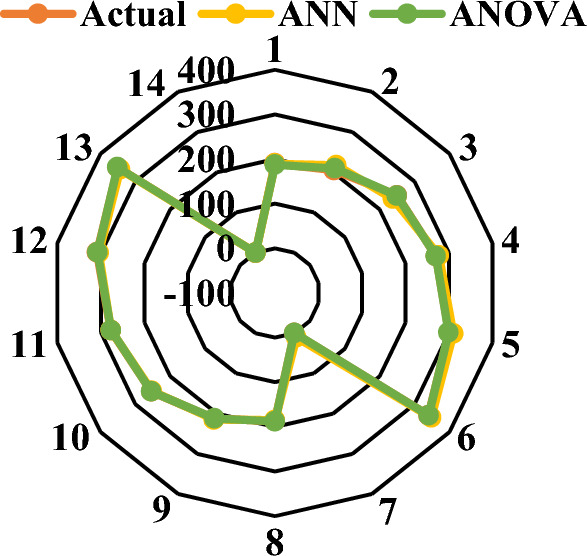
Actual and predicted value of water absorption.

The water absorption R coefficient of correlation was 0.99993. Both parameters are in the range of R correlation coefficients above 0.9 show that the ANN method produces suitable models (Fig. 10). Table 8 displays the outcomes of the experiments and the models used to estimate the water absorption. Again, the results both models' predictions are in agreement results observed during the research, as seen in the Fig. 11 below.

**Table 8:** Actual and predicted value of ANOVA and RSM for water absorption of RHCB.

Mix design	Water absorption		
	Actual	ANN	ANOVA
RH1	190	189.481	187.05
RH2	205	217.472	209.54
RH3	250	238.059	249.43
RH4	270	276.991	269.74
RH5	300	310.059	298.86
RH6	340	347.843	340.38
RH7	0	10.5363	− 0.0106
RHFH1	187	184.946	187.79
RHFH2	213	215.213	212.32
RHFH3	254	252.581	254.23
RHFH4	277	276.777	276.64
RHFH5	309	304.447	307.72
RHFH6	350	343.706	351.29
RHFH7	0	− 0.2562	0.0119

**Figure 12:**
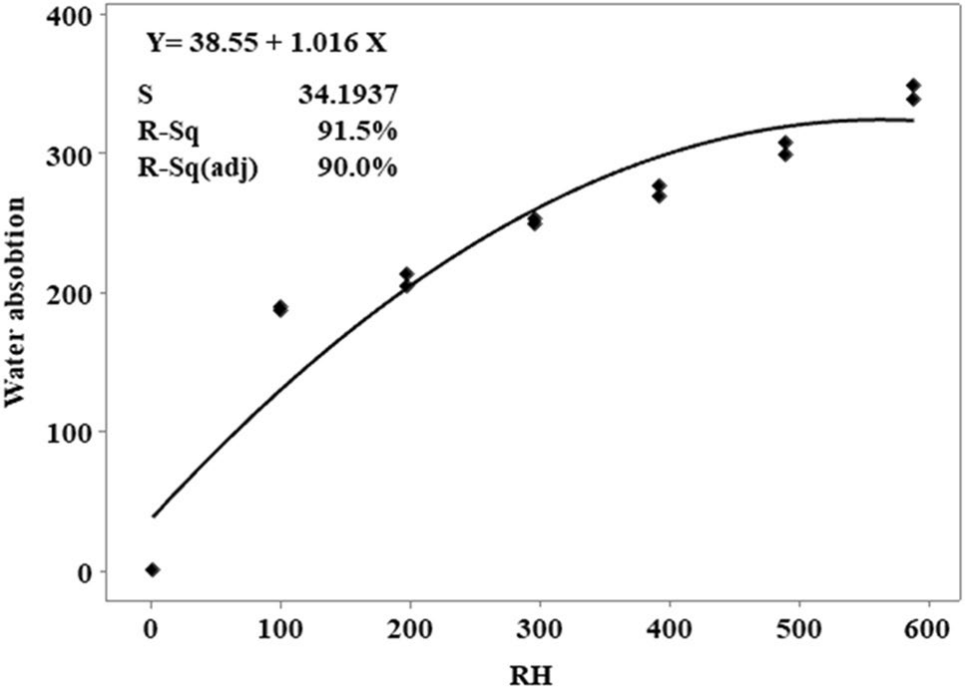
MLR for water absorption. Note* Water absorption (%), RH (g).

Graphic depiction of a radar system in Fig. 11 shows the model’s results compared to the test’s results. To put it another way, the values estimated by the outcomes of the examinations are very close. The significance of the results was determined by performing an ANOVA of water absorption models and model terms at a 95% confidence level. And Fig. 12 shows the MLR for water absorption.

Predicted outcomes differ greatly from actual outcomes of water absorption comparing between the MLR model and the results obtained in the laboratory (Figs. 11, 12). In Fig. 12, the higher RH indicates the higher the water absorption in RHCB. The data points are fitted line plots in Fig. 11. According to the MLR, 0.915 R^2^ predicts the outcomes correctly at 91.5% for water absorption.

### Density

Based on RSM methods and 14 experiments, the 28-day density was created. A quadratic model is used to create it. The density of RHCB results are shown in Table 9.

**Table 9:** AVOVA of density.

Source	Sum of squares	*df*	Mean square	F-value	*p* Value	
Model	152,200	8	19,018.93	2660.97	< 0.0001	Significant
A-C	0.4246	1	0.4246	0.0594	0.8171	
B-D	158.45	1	158.45	22.17	0.0053	
C-E	157.42	1	157.42	22.02	0.0054	
AB	0.4631	1	0.4631	0.0648	0.8092	
AC	0.0402	1	0.0402	0.0056	0.9432	
BC	158.65	1	158.65	22.2	0.0053	
B^2^	159.48	1	159.48	22.31	0.0052	
C^2^	157.73	1	157.73	22.07	0.0053	
Residual	35.74	5	7.15			
Cor total	152,200	13				
SD	2.67	R^2^	0.9998	Adeq precision	163.8869	
Mean	224.64	Adjusted R^2^	0.9994			
C.V.%	1.19	Predicted R^2^	0.9972			

**Figure 13:**
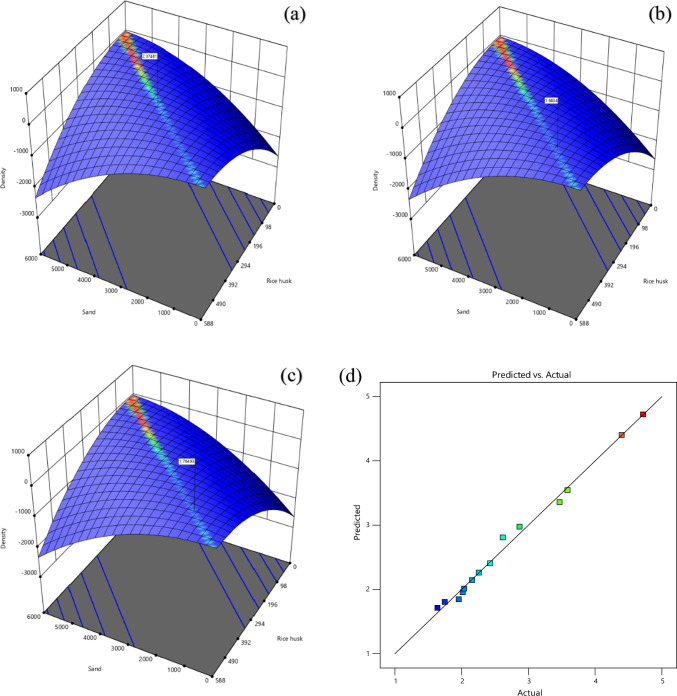
Maximum density, optimum density and minimum density and actual and predicted value from RSM.

The model F-quantity of 86.46 indicates that the model is significant. And the F-quantity greater than 0.01% is extremely unlikely to occur due to noise. A model has significant terms if its P-quantity is less than 0.500. If the value is greater than 0.1000, the model term is unacceptable. Reducing the number of unimportant model term (excluding those required to support hierarchy) can help the model perform better. Because the predicted R^2^ is 0.9429 and the adjusted R^2^ is 0.9813, the two are within 0.2 of each other. Model precision is the ratio of signal to noise. More than a four-to-one ratio is preferable. A signal strength ratio of 28.085 indicates that the data is adequate. The design space can be navigated with the help of this model (Table 9). The maximum Density was calculated to be 2.37481, the Minimum was calculated to be 1.76493, and the target was calculated to be 1.6834 according to this Fig. 13.

**Figure 14:**
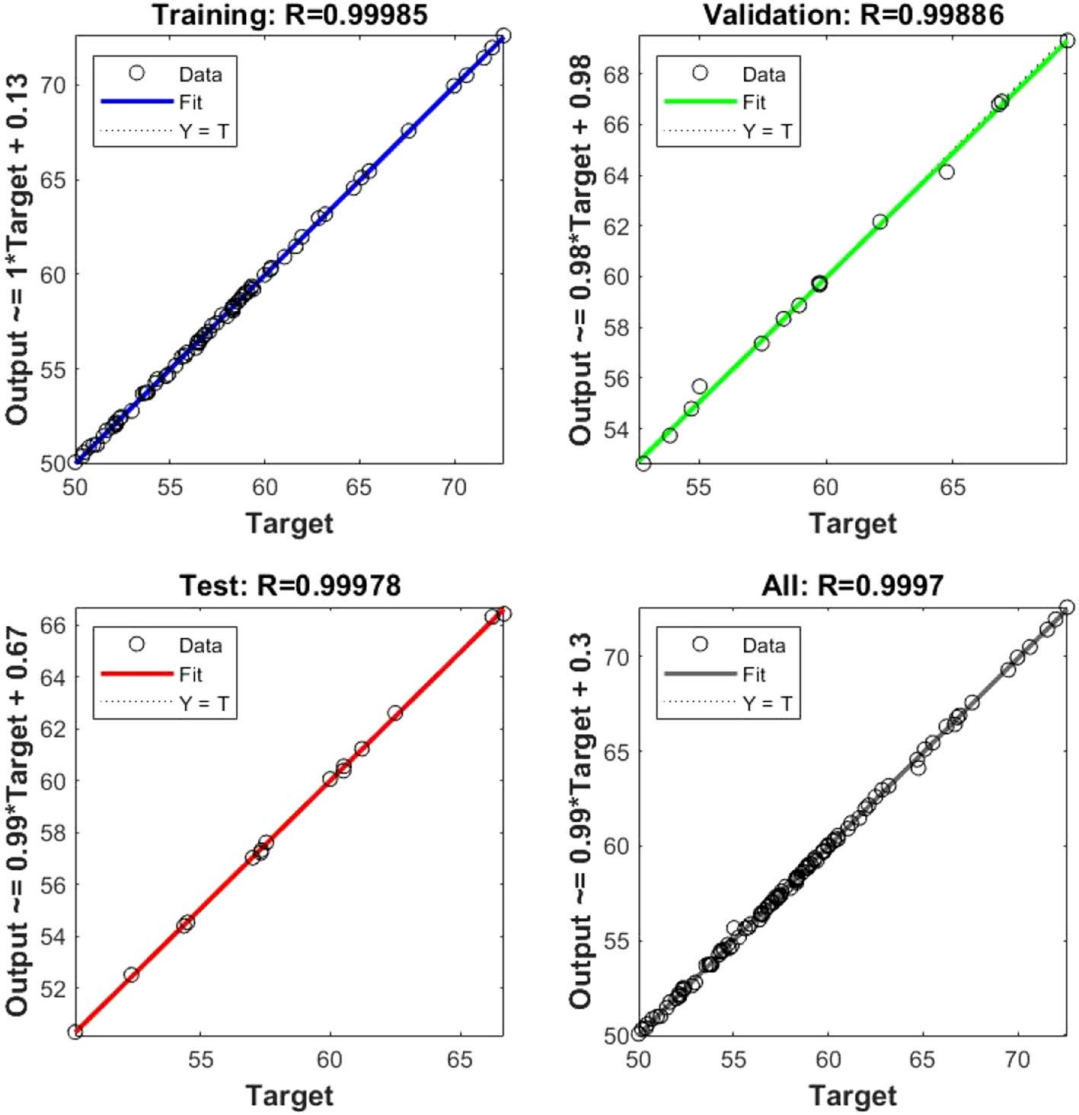
Density with ANN.

The closeness of the actuality and forecast responses can be seen in the response values, which compare the actual with the predicted. The residual plots showed no significant deviation from normality. Equation (7) is the model equations derived using the RSM approach for density. Figure 14 show the R correlation coefficient for density.

7 $$\begin{aligned} Density & = 2616.59961 + 0.005563{\text{C}} + 0.839868{\text{D}} + 8.71111{\text{E}} \\ & \quad - 7.50{\text{E}} - 07{\text{C*D}} - 8.95{\text{E}} - 06{\text{C}} \times {\text{E}} - 0.001399{\text{D }} \\ & \quad \times {\text{E}} - 0.000067{\text{D}}^{2} - 0.007242{\text{E}}^{2} \\ \end{aligned}$$

**Figure 15:**
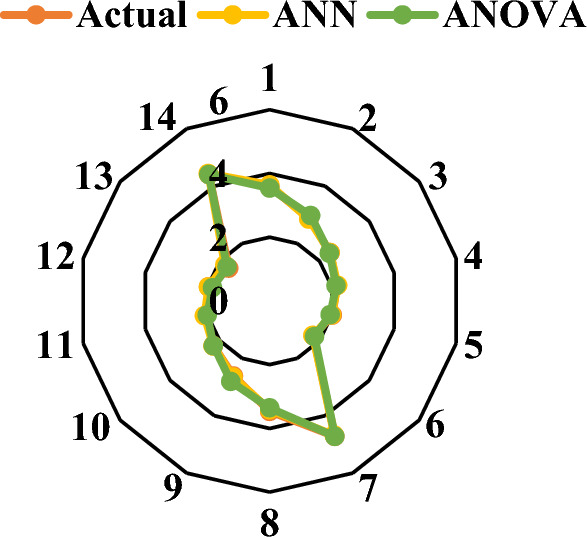
Actual and predicted value of water absorption.

**Figure 16:**
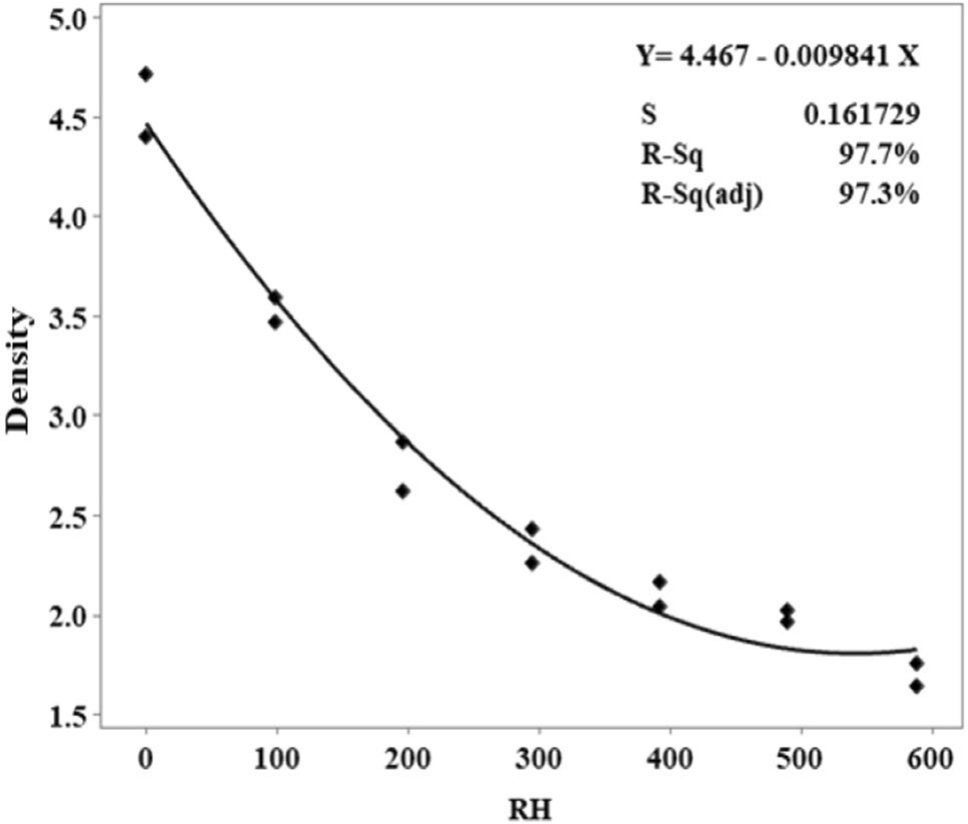
MLR for water absorption.

Table 10 shows the actual and predicted value of ANOVA and RSM for density of RHCB. Following figures (Figs. 15, 16) show the selected model accurately predicted the density and interactions of the materials used.

**Table 10:** Actual and predicted value of ANOVA and RSM for density of RHCB.

Mix design	Density		
	Actual	ANN	ANOVA
RH1	3.59	3.64128	3.54
RH2	2.87	2.84367	2.97
RH3	2.43	2.42373	2.4
RH4	2.16	2.18827	2.14
RH5	2.02	1.99538	1.95
RH6	1.75	1.73463	1.8
RH7	4.72	4.67285	4.72
RHFH1	3.47	3.41633	3.36
RHFH2	2.62	2.65247	2.81
RHFH3	2.26	2.26734	2.26
RHFH4	2.04	2.10022	2.01
RHFH5	1.96	1.96617	1.84
RHFH6	1.64	1.80009	1.71
RHFH7	4.4	4.43694	4.4

Predicted outcomes differ greatly from actual outcomes of density values, comparing between the MLR model and the results obtained in the laboratory. In Fig. 14, the higher RH indicates the lower density. The data points are fitted line plots in Fig. 15. According to the MLR, 0.977 R^2^ predicts the outcomes correctly at 97.7% for density of RHBC.

## Performance analysis

Calculated compressive strength, water absorption, and density of RHCB is compared against those obtained through laboratory testing as part of the performance analysis. Performance is evaluated using four indicators the accuracy of the equations used in the study. The MAE^72^, RMSE^73^, the VAF^74^, and R^2^^75, 76^ are used to evaluate compressive strength, water absorption, and density prediction. When comparing two estimates, MAE measures the difference between the estimates based on each method. The regular equation is shown in Eq. (8).

8 $$MAE= \frac{{\sum }_{i=1}^{n}\left|{y}_{i}-{x}_{i}\right|}{n}$$

where in the analysis, the y_i_ is prediction, the x_i_ is true value, and the n is total number of data points from laboratory experiments and a model that was based on those results. With the RMSE, one can compare the accuracy of different dataset models. Measures the further estimated values stray from actual measurements^77, 78^. RMSE is calculated using the following Eq. (9).

9 $$RMSE=\sqrt{\frac{{\sum }_{i=1}^{N}{\left({x}_{i}-{\hat{u} }_{i}\right)}^{2}}{N}}$$

where the *i* is variable, the *N* is number of non-missing data points, the *x*_*i*_ is actual observations time series, and the *û* is an estimated time series. This statistic is almost always positive, indicating that the model's data has been perfectly fitted when it is equal to zero. A high VAF indicates better forecasting performance for a given dataset since VAF is an indicator of the precision of a prediction method^79^. Calculating VAF is a simple process Eq. (10).

10 $$VAF=\left(1-Var\left({\theta }_{test}-{\theta }_{test}\right)Var ({\theta }_{test})\right)\times 100$$

Var denotes an estimate of the variance in a set of data, VAF is a common verification technique, as well the model’s correctness through comparison of values that have been observed or measured are compared to values that have been predicted or estimated. A model’s prediction performance improves if its MAE and RMSE are lower, and the opposite is true. R^2^ and VAF values, on the other hand, have a direct impact on the accuracy of the model's predictions. A property’s R^2^ is typically calculated by plotting its measured and estimated data. MAE and RMSE are error measures for parameter estimations. As opposed to this, R^2^ and VAF attempt to gauge how close the predicted and measured values of two variables are to each other^80^.

### Sensitive analysis

Sensitivity analysis (SA) to ascertain the individual influence of input parameters on outcomes^81^. SA is executed through technical Eqs. (11) and (12) to quantify the relative contributions of each parameter.

11 $${N}_{i}={f}_{max}\left({x}_{i}\right)-{f}_{min}\left({x}_{i}\right)$$

12 $$SA=\frac{{N}_{i}}{{\sum }_{n}^{j=i}{N}_{j}}$$

${f}_{min}\left({x}_{i}\right)$= predicted model (minimum output)

$$f_{min} \left( {x_{i} } \right) = {\text{predicted}}\;{\text{model }}\left( {{\text{maximum}}\;{\text{output}}} \right)$$

i = expressing the domain of the input variables while holding other variables constant.

**Figure 17:**
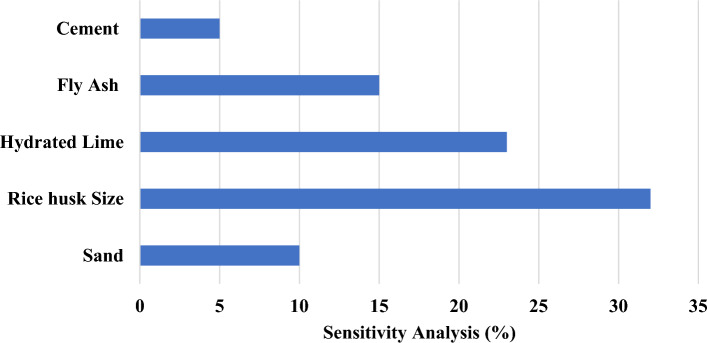
Sensitive analysis.

As depicted in Fig. 17, every parameter holds substantial importance in predicting compressive strength, water absorption, and dry density. The sensitivity analysis underscores the pronounced influence of Rice husk on the actual contributions to compressive strength, water absorption, and dry density, with an impact exceeding 30%. This finding accentuates the significance of Rice husk as a pivotal factor in determining these material properties within the context of the study^82^.

## Discussion

The results stemming from this research can be attributed to the comprehensive utilization of advanced techniques for optimizing raw rice husk-concrete brick (RHCB) properties. The systematic replacement of raw rice husk, fly ash, and hydrated lime for fine aggregate and cement was judiciously evaluated, forming the foundation for robust material compositions. The employment of response surface methodology (RSM) facilitated the exploration of intricate relationships between variables, leading to the precise optimization of compressive strength, water absorption, and dry density. The model's subsequent validation through artificial neural network (ANN) and Multiple Linear Regression showcased its efficacy across varied scenarios, as indicated by the high R and R^2^ thresholds achieved^83^.

The exceptional accuracy attained in predicting 28-day compressive strength, water absorption, and density can be attributed to the synergistic interplay between RSM and ANN, outperforming Multiple Linear Regression. The lower MAE and RMSE values, along with elevated R^2^ metrics, definitively underscore the model's exceptional predictive capabilities. The validation of outcomes via sensitivity analysis further bolsters the results, enabling a granular understanding of the input parameter influences. The culmination of these techniques, encapsulating machine learning, optimization, and rigorous analysis, presents a sound scientific approach to enhancing concrete's mechanical properties, while streamlining resource utilization in the domain of civil engineering.

ANN, RSM, and MLR models were identified as contributing factors to predicting CS, density, and water absorption values. In compressive strength, the MAE, RMSE, VAF, and R^2^ are the performance indicators compared. The ANN model predicts an MAE of 0.031, RMSE of 0.361, R^2^ of 0.99997, and VAF of 99.32%. The MAE predicted by the RSM model was 0.097, the RMSE was 0.126, the R^2^ was 0.9923, and the VAF was 99.23%, while the MAE predicted by the MLR model was 0.038, the RMSE was 0.392, the R^2^ was 0.9855, and the VAF was 98.55%. The MAE, RMSE, R^2^, and VAF are used for density comparisons. In the ANN model, the MAE was 0.039, RMSE was 0.054, R^2^ was 0.99993, and VAF was 99.85%, while, in the RSM model, it was 0.06, RMSE was 0.080, R^2^ was 0.9998, and VAF was 99.99%, while in the MLR model it was 0.026, RMSE was 1.283, R^2^ was 0.9768, and VAF was 96.78%. Indicators of performance that are being compared in water absorption are the MAE, RMSE, R^2^, and VAF. The ANN model yielded an MAE of 5,526, an RMSE of 7,033, an R^2^ of 0.99997, and a VAF of 99.85. The MAE of the RSM model was 1.035, the RMSE was 1.597, R^2^ was 0.9998, and the VAF was 99.98. The MAE of the MLR model was 0.934, the RMSE was 9.753, R^2^ was 0.9155, and the VAF was 91.55. Table 11 shows, based on the evaluation of performance, this signifies that the ANN, RSM model is a better an algorithm for predicting the future CS, density, and water absorption than the MLR model.

**Table 11:** ANN, MLR, and RSM comparison model.

Statistical indices	Compressive strength models			Density models			Water absorption models		
	ANN	MLR	RSM	ANN	MLR	RSM	ANN	MLR	RSM
MAE	0.031	0.038	0.097	0.039	0.026	0.06	5.526	0.934	1.035
RMSE	0.361	0.392	0.126	0.054	1.283	0.080	7.033	9.753	1.597
R^2^	0.999	0.9855	0.9923	0.999	0.9768	0.9998	0.9993	0.9155	0.9998
VAF(%)	99.32	98.55	99.23	99.85	97.68	99.99	99.83	91.55	99.98

The MLR model’s lower performance may be due to the model’s lack of completeness depict and account for the uncertainties in the dataset's input. Because of these uncertainties, the MLR model's performance will be lower. The ANN, RSM model's training algorithm quantifies the inherent uncertainties in the input data, resulting in a better model. According to MLR's predicted compressive and water absorption and density were slightly overestimated compared to the actual measurements. The above signifies that while the MLR can be used as a model estimate RHCB's compressive strength, density, and water absorption properties, ANN and RSM models are more accurate at predicting RHCB's compressive strength, water absorption, and density properties (Table 11).

### Practical applications

In hot climates, ANN models can assist construction site engineers in selecting the most appropriate concrete parameters, resulting more durable and long-lasting construction materials^84^. The results have numerous useful applications for engineers in the fields of civil and environmental construction. The use of these types of design models is important for estimating the performance of a product on the basis of a predetermined objective cement substitutes made from other types of solid waste^85^. As a result of the surrounding benefits of cement that has been reduced consumption, careful consideration must be given to the decision to use a partial replacement, as must maintaining a minimum level of mechanical performance beginning for concrete as mandated by regulation^86^. Predictive models are applicable and helpful in such circumstances. In addition, concerns about costs, concerns about the budget, and in the beginning, cost estimates for engineering of the built environment projects can benefit from such prediction models. In comparison to theoretical and experimental methods, RSM, ANN and MLR predictions are rapid and less costly^87^.

## Conclusion

The compressive strength, water abortion, and density of RHCB were predicted using ANN, RSM, and MLR models in this current work. The models took as input three quantitative parameters like raw rice husk, FA, and hydrated lime. Compressive strength, water absorption, and density were studied in relation to RH, FA, and HL using ANN, RSM, and MLR methods. The results are as follows:

- As the percentage of raw rice husk replaced increased, a decrease in strength was observed in the RHCB. Replacing rice husk with sand 1:3:3 is significant water absorption and density noted according to the literature.
- R^2^ values for compressive, water abortion, and density were 0.9923, 0.9998, and 0.9998, respectively, after RSM analysis. Therefore, the models estimate the compression strength to be 99.23%, the water abortion to be 99.98%, and the density to be 99.98% based on these obtained values.
- The values of compressive strength, water abortion, density, and R correlation coefficients were 0.9939, 0.99839 and 0.99875, respectively. The ANN model was appropriate because all correlation coefficients are nearly close to 1.
- R^2^ values for CS, water abortion, and density were 0.9855, 0.9155, and 0.9768, respectively, after MLR analysis. Therefore, the models estimate the compression strength to be 98.55%, the water abortion to be 91.55%, and the density to be 97.68% based on these obtained values.
- A comparison of plots of parity showed that both the ANN and RSM are performing models had no bias in their prediction accuracy. The RSM, ANN model, on the other hand, is superior to the MLR model because it is more accurate and better suited to the dataset.

## Data Availability

The datasets generated and analyzed during the current study are available from the corresponding author on reasonable request.

## Abbreviations

RH: Rice husk
HL: Hydrated lime
FA: Fly ash
RSM: Response surface methodology
ANN: Artificial neural network
MLR: Multiple linear regression
SS: Sum of squares
MS: Mean square
MAE: Mean absolute error
RMSE: Root mean squared error
VAF: Variance accounted for
ANOVA: Analysis of variance

## References

[1] Shafigh P, Mahmud HB, Jumaat MZ, Zargar M (2014). Agricultural wastes as aggregate in concrete mixtures—A review. Constr. Build. Mater..

[2] Santhosh KG, Subhani SM, Bahurudeen A (2022). Recycling of palm oil fuel ash and rice husk ash in the cleaner production of concrete. J. Clean. Prod..

[3] Krishnaraj L, Niranjan R, Kumar GP, Kumar RS (2020). Numerical and experimental investigation on mechanical and thermal behaviour of brick masonry: An efficient consumption of ultrafine fly ash. Constr. Build. Mater..

[4] U.S. Department of Agriculture | USAGov. https://www.usa.gov/federal-agencies/u-s-department-of-agriculture.

[5] Siddiqui ZA, Khan MR, Ahamad L (2022). Effects of fly ash on growth, productivity, and diseases of crop plants. Handb. Fly Ash.

[6] Hlobil M, Sotiriadis K, Hlobilová A (2022). Scaling of strength in hardened cement pastes—Unveiling the role of microstructural defects and the susceptibility of C–S–H gel to physical/chemical degradation by multiscale modeling. Cem. Concr. Res..

[7] Luukkonen T, Abdollahnejad Z, Yliniemi J, Kinnunen P, Illikainen M (2018). One-part alkali-activated materials: A review. Cem. Concr. Res..

[8] Krishnya S (2022). Modeling of hydration products and strength development for high-volume fly ash binders. Constr. Build. Mater..

[9] Nakkeeran G, Krishnaraj L (2023). Prediction of cement mortar strength by replacement of hydrated lime using RSM and ANN. Asian J. Civ. Eng..

[10] Prusty JK, Patro SK, Basarkar SS (2016). Concrete using agro-waste as fine aggregate for sustainable built environment—A review. Int. J. Sustain. Built Environ..

[11] Alghamdi H (2022). A review of cementitious alternatives within the development of environmental sustainability associated with cement replacement. Environ. Sci. Pollut. Res..

[12] Maderuelo-Sanz R (2022). Mechanical, thermal and acoustical evaluation of biocomposites made of agricultural waste for ceiling tiles. Appl. Acoust..

[13] Sabino TPF (2022). Lignocellulosic materials as soil–cement brick reinforcement. Environ. Sci. Pollut. Res..

[14] Borges JK, Pacheco F, Tutikian B, de Oliveira MF (2018). An experimental study on the use of waste aggregate for acoustic attenuation: EVA and rice husk composites for impact noise reduction. Constr. Build. Mater..

[15] Ketov A (2021). Amorphous silica wastes for reusing in highly porous ceramics. Int. J. Appl. Ceram. Technol..

[16] Srivastava M, Kumar V (2018). The methods of using low cost housing techniques in India. J. Build. Eng..

[17] Ganasen N, Bahrami A, Loganathan K (2023). A scientometric analysis review on agricultural wastes used as building materials. Buildings.

[18] Narattha C, Wattanasiriwech S, Wattanasiriwech D (2022). Thermal and mechanical characterization of fly ash geopolymer with aluminium chloride and potassium hydroxide treated hemp shiv lightweight aggregate. Constr. Build. Mater..

[19] B. of Indian Standards. IS 2185-1. Concrete masonry units, Part 1: Hollow and solid concrete blocks (2005).

[20] Sapkota SC, Saha P, Das S, Meesaraganda LP (2023). Prediction of the compressive strength of normal concrete using ensemble machine learning approach. Asian J. Civ. Eng..

[21] Onyelowe KC (2023). The influence of fines on the hydro-mechanical behavior of sand for sustainable compacted liner and sub-base construction applications. Asian J. Civ. Eng..

[22] Tipu RK, Batra V (2023). Enhancing prediction accuracy of workability and compressive strength of high-performance concrete through extended dataset and improved machine learning models. Asian J. Civ. Eng..

[23] Feng DC (2020). Machine learning-based compressive strength prediction for concrete: An adaptive boosting approach. Constr. Build. Mater..

[24] Xu J (2019). Parametric sensitivity analysis and modelling of mechanical properties of normal- and high-strength recycled aggregate concrete using grey theory, multiple nonlinear regression and artificial neural networks. Constr. Build. Mater..

[25] Golafshani EM, Behnood A (2018). Application of soft computing methods for predicting the elastic modulus of recycled aggregate concrete. J. Clean. Prod..

[26] Getahun MA, Shitote SM, Abiero Gariy ZC (2018). Artificial neural network based modelling approach for strength prediction of concrete incorporating agricultural and construction wastes. Constr. Build. Mater..

[27] Hammoudi A, Moussaceb K, Belebchouche C, Dahmoune F (2019). Comparison of artificial neural network (ANN) and response surface methodology (RSM) prediction in compressive strength of recycled concrete aggregates. Constr. Build. Mater..

[28] Asteris PG, Mokos VG (2020). Concrete compressive strength using artificial neural networks. Neural Comput. Appl..

[29] Dantas ATA, Batista Leite M, De Jesus Nagahama K (2013). Prediction of compressive strength of concrete containing construction and demolition waste using artificial neural networks. Constr. Build. Mater..

[30] Golafshani EM, Behnood A, Arashpour M (2020). Predicting the compressive strength of normal and high-performance concretes using aNN and ANFIS hybridized with Grey Wolf Optimizer. Constr. Build. Mater..

[31] Chithra S, Kumar SRRS, Chinnaraju K, AlfinAshmita F (2016). A comparative study on the compressive strength prediction models for High performance concrete containing nano silica and copper slag using regression analysis and Artificial Neural Networks. Constr. Build. Mater..

[32] Bui DK, Nguyen T, Chou JS, Nguyen-Xuan H, Ngo TD (2018). A modified firefly algorithm-artificial neural network expert system for predicting compressive and tensile strength of high-performance concrete. Constr. Build. Mater..

[33] Moradi MJ, Khaleghi M, Salimi J, Farhangi V, Ramezanianpour AM (2021). Predicting the compressive strength of concrete containing metakaolin with different properties using ANN. Measurement.

[34] Naderpour H, Rafiean AH, Fakharian P (2018). Compressive strength prediction of environmentally friendly concrete using artificial neural networks. J. Build. Eng..

[35] Rezaifar O, Hasanzadeh M, Gholhaki M (2016). Concrete made with hybrid blends of crumb rubber and metakaolin: optimization using response surface method. Constr. Build. Mater..

[36] Sun Y (2019). Understanding the porous aggregates carrier effect on reducing autogenous shrinkage of ultra-high performance concrete (UHPC) based on response surface method. Constr. Build. Mater..

[37] Ferdosian I, Camões A (2017). Eco-efficient ultra-high performance concrete development by means of response surface methodology. Cem. Concr. Compos..

[38] Elemam WE, Abdelraheem AH, Mahdy MG, Tahwia AM (2020). Optimizing fresh properties and compressive strength of self-consolidating concrete. Constr. Build. Mater..

[39] Boukli Hacene SMA, Ghomari F, Schoefs F, Khelidj A (2014). Probabilistic modelling of compressive strength of concrete using response surface methodology and neural networks. Arab. J. Sci. Eng..

[40] Shakr Piro N, Mohammed A, Hamad SM, Kurda R (2022). Electrical resistivity—Compressive strength predictions for normal strength concrete with waste steel slag as a coarse aggregate replacement using various analytical models. Constr. Build. Mater..

[41] Patil, S. V., Balakrishna Rao, K. & Nayak, G. Prediction of recycled coarse aggregate concrete mechanical properties using multiple linear regression and artificial neural network. *J. Eng. Des. Technol.* (2021).

[42] Amiri H, Azadi S, Karimaei M, Sadeghi H, Dabbaghi F (2022). Multi-objective optimization of coal waste recycling in concrete using response surface methodology. J. Build. Eng..

[43] Wang X, Yu R, Shui Z, Song Q, Zhang Z (2017). Mix design and characteristics evaluation of an eco-friendly Ultra-High Performance Concrete incorporating recycled coral based materials. J. Clean. Prod..

[44] Nakkeeran G (2023). Machine learning application to predict the Mechanical properties of Glass Fiber mortar. Adv. Eng. Softw..

[45] Kursuncu B (2022). Optimization of foam concrete characteristics using response surface methodology and artificial neural networks. Constr. Build. Mater..

[46] Mosaberpanah MA, Eren O, Tarassoly AR (2019). The effect of nano-silica and waste glass powder on mechanical, rheological, and shrinkage properties of UHPC using response surface methodology. J. Market. Res..

[47] Iftikhar B (2023). Predicting compressive strength of eco-friendly plastic sand paver blocks using gene expression and artificial intelligence programming. Sci. Rep..

[48] Chen Z (2023). Predictive modelling for the acid resistance of cement-based composites modified with eggshell and glass waste for sustainable and resilient building materials. J. Build. Eng..

[49] Iftikhar B (2023). Experimental study on the eco-friendly plastic-sand paver blocks by utilising plastic waste and basalt fibers. Heliyon.

[50] Nasir Amin M (2023). Prediction model for rice husk ash concrete using AI approach: Boosting and bagging algorithms. Structures.

[51] Albostami AS, Al-Hamd RKS, Alzabeebee S, Minto A, Keawsawasvong S (2023). Application of soft computing in predicting the compressive strength of self-compacted concrete containing recyclable aggregate. Asian J. Civ. Eng..

[52] Kaveh A, Servati H (2001). Design of double layer grids using backpropagation neural networks. Comput. Struct..

[53] Kaveh, A., Servati, H. & Fazel, D. Prediction of moment-rotation characteristic for saddle-like connections using FEM and BP neural networks (2001).

[54] Kaveh, A., Elmieh, R. & Servati, H. Prediction of moment-rotation characteristic for semi-rigid connections using BP neural networks (2001).

[55] Rajamane NP, Annie Peter J, Ambily PS (2007). Prediction of compressive strength of concrete with fly ash as sand replacement material. Cem. Concr. Compos..

[56] Hatungimana D, Taşköprü C, İçhedef M, Saç MM, Yazıcı Ş (2019). Compressive strength, water absorption, water sorptivity and surface radon exhalation rate of silica fume and fly ash based mortar. J. Build. Eng..

[57] Khalid FS (2021). Density, compressive strength and water absorption properties of sand cement brick containing recycled concrete aggregate (RCA) and crumb rubber (CR) as partial sand replacement materials. Lect. Notes Civ. Eng..

[58] Sathiparan N, de Zoysa HTSM (2018). The effects of using agricultural waste as partial substitute for sand in cement blocks. J. Build. Eng..

[59] Khuri AI, Mukhopadhyay S (2010). Response surface methodology. Wiley Interdiscip. Rev. Comput. Stat..

[60] Ighalo JO, Adelodun AA, Adeniyi AG, Igwegbe CA (2020). Modelling the effect of sorbate-sorbent interphase on the adsorption of pesticides and herbicides by historical data design. Iran. (Iran.) J. Energy Environ..

[61] Ekpotu WF, Ighalo JO, Nkundu KAB, Onu PO, Adeniyi AG (2020). Analysis of factor effects and interactions in a conventional drilling operation by response surface methodology and historical data design. Pet. Coal.

[62] Parichatprecha R, Nimityongskul P (2009). Analysis of durability of high performance concrete using artificial neural networks. Constr. Build. Mater..

[63] AlanemeGeorge U, Elvis M (2019). Modelling of the mechanical properties of concrete with cement ratio partially replaced by aluminium waste and sawdust ash using artificial neural network. SN Appl. Sci..

[64] Dahmoune F (2015). Ultrasound assisted extraction of phenolic compounds from *P. lentiscus* L. leaves: Comparative study of artificial neural network (ANN) versus degree of experiment for prediction ability of phenolic compounds recovery. Ind. Crops Prod..

[65] Alaneme GU, Onyelowe KC, Onyia ME, Bui Van D, Mbadike EM, Ezugwu CN, Dimonyeka MU, Attah IC, Ogbonna C, Abel C, Ikpa CC, Udousoro IM (2020). Modeling volume change properties of hydrated-lime activated rice husk ash (HARHA) modified soft soil for construction purposes by artificial neural network (ANN). Umudike J. Eng. Technol..

[66] Said KO, Onifade M, Lawal AI, Githiria JM (2021). An artificial intelligence-based model for the prediction of spontaneous combustion liability of coal based on its proximate analysis. Combust. Sci. Technol..

[67] Alaneme GU, Mbadike EM (2021). optimization of strength development of bentonite and palm bunch ash concrete using fuzzy logic. Int. J. Sustain. Eng..

[68] Ustaoglu A, Kursuncu B, Alptekin M, Engineering MGAT (2020). Performance optimization and parametric evaluation of the cascade vapor compression refrigeration cycle using Taguchi and ANOVA methods.

[69] Chaliha C, Kalita E, Verma PK (2020). Optimizing In vitro culture conditions for the biotrophic fungi *Exobasidium vexans* through response surface methodology. Indian J. Microbiol..

[70] Alaneme GU, Mbadike EM, Attah IC (2022). Udousoro IM (2022) Mechanical behaviour optimization of saw dust ash and quarry dust concrete using adaptive neuro-fuzzy inference system. Innov. Infrastruct. Solut..

[71] IbeIro U, Alaneme GU, Milad A, Olaiya BC, Otu ON, Isu EU, Amuzie MN (2022). Optimization and simulation of saw dust ash concrete using extreme vertex design method. Adv. Mater. Sci. Eng..

[72] de Myttenaere A, Golden B, le Grand B, Rossi F (2016). Mean absolute percentage error for regression models. Neurocomputing.

[73] Karunasingha DSK (2022). Root mean square error or mean absolute error? Use their ratio as well. Inf. Sci. (N. Y.).

[74] Mohamed Shabeer KP, Unni Krishnan SI, Deepa G (2022). Software effort estimation using genetic algorithms with the variance-accounted-for (VAF) and the Manhattan distance. Smart Innovat. Syst. Technol..

[75] Ighalo JO, Adeniyi AG, Marques G. 2020. Application of artificial neural networks in predicting biomass higher heating value: an early appraisal. *Energy Sources Part A: Recov. Util. Environ. Effects* (2020) 10.1080/15567036.2020.1809567.

[76] Adeniyi AG, Igwegbe CA, Ighalo JO (2021). ANN modelling of the adsorption of herbicides and pesticides based on sorbate-sorbent interphase. Chem. Afr..

[77] Hyndman RJ, Koehler AB (2005). Another look at measures of forecast accuracy. Int. J. Forecast..

[78] Ewa DE, Ukpata JO, Otu ON, Memon ZA, Alaneme GU, Milad A (2023). Scheffe’s simplex optimization of flexural strength of quarry dust and sawdust ash pervious concrete for sustainable pavement construction. Materials.

[79] Bahaaddini M, Hosseinpour Moghadam E (2019). Evaluation of empirical approaches in estimating the deformation modulus of rock masses. Bull. Eng. Geol. Environ..

[80] Agor CD, Mbadike EM, Alaneme GU (2023). Evaluation of sisal fiber and aluminum waste concrete blend for sustainable construction using adaptive neuro-fuzzy inference system. Sci. Rep..

[81] Iftikhar B (2023). A machine learning-based genetic programming approach for the sustainable production of plastic sand paver blocks. J. Market. Res..

[82] Ewa DE, Ukpata JO, Out ON, Alaneme GU (2023). Optimization of saw dust ash and quarry dust pervious concrete’s compressive strength using Scheffe’s simplex lattice method. Innov. Infrastruct. Solut..

[83] Alaneme GU, Mbadike EM, Iro UI, Udousoro IM, Ifejimalu WC (2021). Adaptive neuro-fuzzy inference system prediction model for the mechanical behaviour of rice husk ash and periwinkle shell concrete blend for sustainable construction. Asian J. Civ. Eng..

[84] Maqsoom A (2021). Using multivariate regression and ANN models to predict properties of concrete cured under hot weather. Sustainability.

[85] Alaneme GU (2022). Mechanical strength optimization and simulation of cement kiln dust concrete using extreme vertex design method. Nanotechnol. Environ. Eng..

[86] Imbabi MS, Carrigan C, McKenna S (2012). Trends and developments in green cement and concrete technology. Int. J. Sustain. Built Environ..

[87] Shahmansouri AA (2021). Artificial neural network model to predict the compressive strength of eco-friendly geopolymer concrete incorporating silica fume and natural zeolite. J. Clean. Prod..

